# Single-cell evaluation of red blood cell bio-mechanical and nano-structural alterations upon chemically induced oxidative stress

**DOI:** 10.1038/srep09768

**Published:** 2015-05-07

**Authors:** Ameya Sinha, Trang T. T. Chu, Ming Dao, Rajesh Chandramohanadas

**Affiliations:** 1 Engineering Product Development (EPD) Pillar, Singapore University of Technology & Design (SUTD), Singapore; 2 Interdisciplinary Research Group of Infectious Diseases, Singapore MIT Alliance for Research & Technology Centre (SMART), Singapore; 3 Department of Materials Science and Engineering, Massachusetts Institute of Technology, Cambridge-Massachusetts, U.S.A

## Abstract

Erythroid cells, specifically red blood cells (RBCs), are constantly exposed to highly reactive radicals during cellular gaseous exchange. Such exposure often exceeds the cells' innate anti-oxidant defense systems, leading to progressive damage and eventual senescence. One of the contributing factors to this process are alterations to hemoglobin conformation and globin binding to red cell cytoskeleton. However, in addition to the aforementioned changes, it is possible that oxidative damage induces critical changes to the erythrocyte cytoskeleton and corresponding bio-mechanical and nano-structural properties of the red cell membrane. To quantitatively characterize how oxidative damage accounts for such changes, we employed single-cell manipulation techniques such as micropipette aspiration and atomic force microscopy (AFM) on RBCs. These investigations demonstrated visible morphological changes upon chemically induced oxidative damage (using hydrogen peroxide, diamide, primaquine bisphosphate and cumene hydroperoxide). Our results provide previously unavailable observations on remarkable changes in red cell cytoskeletal architecture and membrane stiffness due to oxidative damage. Furthermore, we also demonstrate that a pathogen that infects human blood cells, *Plasmodium falciparum* was unable to penetrate through the oxidant-exposed RBCs that have damaged cytoskeleton and stiffer membranes. This indicates the importance of bio-physical factors pertinent to aged RBCs and it's relevance to malaria infectivity.

Oxidative stress occurs when the cell's antioxidant defense system becomes no longer able to combat the levels of reactive oxygen species (ROS) generated ubiquitously in the body^1^. It is a significant cause of cell and tissue damage and is associated with diseases such as anemia, atherosclerosis and diabetes mellitus^2^. Progressive ageing and senescence in red blood cells (RBCs) are also thought to be a consequence of oxidative damage^3^. Circulating RBCs are particularly susceptible to oxidative stress due to the high content of polyunsaturated fatty acids in their lipid bilayer, continuous exposure to high oxygen levels and the auto-oxidation of hemoglobin leading to heme degradation^4^. Further on, this oxidative damage has been shown to cause clustering of Band-3 anion exchange protein and also cytoskeletal reorganization. These changes alter membrane fluidity, membrane potential, permeability to ions and eventually lead to hemolysis^5^.

Erythrocytes have the remarkable ability to deform thereby aiding their passage through narrow capillaries to facilitate gaseous exchange. Blood flow through the microvasculature is determined by a multitude of factors such as blood vessel geometry and hydrostatic blood pressure^6^. However, reduction in RBC deformability in pathological conditions like sickle cell anemia and malaria infection impairs blood flow through the circulation leading to micro-vascular complications. Various experimental techniques such as rheoscopy, flow channels, ektacytometry and optical trapping^7^ have been used to investigate such alterations to RBC deformability. Each of these methods has unique strengths and limitations based on the type of rheological aspects that are being investigated. Several factors contribute to the deformability of an RBC including shape and size, cell viscosity and membrane rigidity. Techniques such as ektacytometry register population-wide whole cell response to imposed strain^7^. In other words, deformability properties of the whole cells are taken collectively into consideration. Hence, it is not possible to precisely extract individual parameters, such as membrane stiffness (or membrane shear modulus) using ektacytometry.

We have evaluated the effect of hydrogen peroxide, diamide, primaquine bisphosphate and cumene hydroperoxide, four commonly used compounds to inflict oxidative stress *in vitro.* RBC membrane property changes upon oxidative stress were measured through micropipette aspiration method^8^, in combination with nano-structural analyses using atomic force microscopy^9^. Our study demonstrates that different oxidative reagents can alter RBC bio-mechanical properties by causing damage to the structural components to different degrees, as confirmed by atomic force microscopy (AFM). Analysis at the single-cell level has allowed us to examine the response of these RBCs individually subjected to oxidative stress.

## Results

To induce controlled oxidative damage to red cells under experimental conditions, we chose well-studied and representative oxidizing agents, (i) Hydrogen peroxide (H_2_O_2_), (ii) Diamide, (iii) Primaquine bisphosphate (PQ) and (iv) Cumene hydroperoxide (CumOOH). H_2_O_2_ is a water-soluble oxidant readily permeable to cell membranes and is thought to primarily affect the cytoplasmic components^10^. Diamide can function as a thiol-oxidizing reagent and has been a popular candidate to stimulate oxidative stress in several other investigations^11^. PQ was chosen as it mediates hemolysis by the generation of redox-active metabolites and is an antimalarial compound^12^. Finally, CumOOH was included in the studies, as it is known to penetrate into the inner hydrophobic part of the membrane lipid bilayer, causing extensive peroxidation of lipids (to a much greater extent than hydrogen peroxide)^10^.

### Exposure to oxidants induce unique changes to overall RBC morphology and membrane deformability

**Figure 1:**
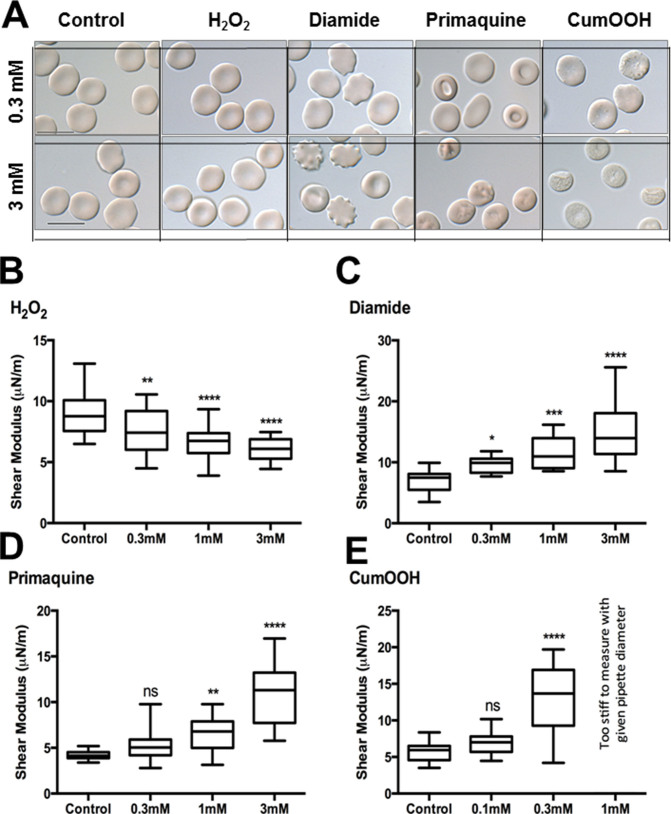
Exposure to oxidants induce global changes to RBC morphology and bio-mechanical properties. (A) DIC images of RBCs treated with different oxidizing agents at varying concentrations demonstrating oxidant-induced morphological changes (Scale bar = 10 µm). (B) Micropipette aspiration results show dose dependent change in the shear modulus of RBCs treated with individual oxidizing agents. Except for H_2_O_2_, all other compounds such as diamide, Primaquine and Cumenehydroperoxide induced changes in the membrane deformability. Data are relative to the corresponding control sample. For H_2_O_2_, an increasing concentration of the compound resulted in decreased shear modulus. Data is represented as median ± range. ns – not significant. * – <0.05, ** – <0.01, *** – <0.001, **** – <0.0001.

Freshly drawn human erythrocytes were treated with different oxidizing agents at different concentrations. All further experimental steps were conducted after thoroughly washing RBCs post oxidant-treatment to solely capture irreversible damages occurred during incubation with various oxidants. First, we conducted microscopic examination to identify apparent changes in basic cellular parameters such as size, shape etc. caused by oxidant exposure (Fig. 1A). Under physiological conditions, a normal human RBC assumes biconcave discoid (discocyte) shape ~ 8 μm in diameter. It is known that the biconcave discoid shape represents an equilibrium state between two opposing extremes of red cell shapes- crenated (echinocytes) and and cup-shaped (stomatocytes) cells. However, from normal RBCs, it is possible to induce either crenated or cup-like cells by anionic/neutral compounds and cationic substances respectively^13^.

Our data indicated that each of the oxidant species used in this study induced unique changes to the normal discocyte shape of RBCs. Fig. 1A shows typical representative phenotypes induced by each compound. Under light microscopy, H_2_O_2_ appeared not to alter the overall RBC morphology (i.e. dimpled biconcave shape), at concentrations of up to 3 mM (Fig. 1A, H_2_O_2_ panel). Treatment with all other agents showed a dose-dependent influence. Diamide transformed the RBCs to echinocytes, characterized by convex rounded protrusions or spicules evenly spaced around the circumference^14^. RBCs treated with PQ, on the other hand, showed a visible change toward stomatocytes, characterized by cup-shaped RBCs with evagination of one surface and a deep invagination of the opposite^15^. This is not surprising since PQ is classically known to induce stomatocytosis^13^. Erythrocytes exposed to 0.3 mM PQ showed stomatocyte-like morphology, whereas 3 mM further converted majority of the cells to knizocytes (RBCs with two or three concavities, separated by a ridge)^16^. Lastly, CumOOH induced rough RBC surfaces with dot-like structures at lower concentration (0.3 mM) and further altered the discocyte to spherocyte at 1 mM (highest concentration of CumOOH used in this study was at 1 mM since 3 mM exposure resulted in complete red cell lysis). The dot-like structures are due to the formation of high-molecular-weight aggregates of proteins (HMWA). These HMWAs remain bound to the membrane^17^. Carpari *et al.*^18^ observed the same phenomenon with the oxidation of RBCs induced by t-BHP, another organic hydroperoxide, in that they showed difficulties of extractability of oxidized spectrin from erythrocyte membranes.

To accurately evaluate possible changes in the membrane deformability of RBCs treated with different oxidizing agents, we adopted the method of micropipette aspiration as described previously^8^. To do this, a reference control value for each donor was established for each set of micropipette experiments, since erythrocytes from different donors may have different healthy shear modulus values. The measurements were carried out only on RBCs that had not lysed or transformed into spiculated echinocytes in response to oxidant treatment. The blood samples we used for analyses comprise of different RBC types normally found in the blood and include discocytes, spherocytes, ovalocytes and stomatocytes. RBCs that had undergone dramatic morphological changes (such as echinocytes and acanthocytes) or lysis were not included in the measurements to avoid artefactual readings. There is no available method (including the micropipette method) yet that can reliably measure RBC membrane shear modulus for spiculated echinocytes, and thus such measurements can introduce significant errors in extracted RBC shear modulus values and were consequently omitted from analyses.

Sufficient number of freshly drawn and washed healthy RBCs were examined as the first step, to obtain a baseline average shear modulus. The detailed values and statistics for each of the micropipette aspiration measurement are listed in the supplementary: S1. Precisely, shear moduli of *μ* = 8.89 µN/m (H_2_O_2_), *μ* = 7.11 µN/m (Diamide), *μ* = 4.19 µN/m (PQ), *μ* = 5.78 µN/m (CumOOH), were obtained as the reference control groups (Fig. 1B–E). These values are consistent for healthy RBC stiffness measurements based on micropipette aspiration^19^ and optical tweezers^20^. All of the compounds, except H_2_O_2_, caused dose dependent increase in the membrane shear modulus relative to the corresponding reference control sample (Fig. 1C–E). The shear moduli corresponding to the highest compound concentrations are as follows: *μ* = 6.05 µN/m (3mM H_2_O_2_), *μ* = 14.92 µN/m (3mM Diamide), *μ* = 10.94 µN/m (3mM PQ), and *μ* = 13.20 (0.3mM CumOOH). The shear moduli for 1mM CumOOH could not be measured (Fig. 1E), as a sufficiently large pressure could not be achieved using the micropipette with the given diameter for these significantly stiffened RBCs.

It is interesting to observe that, increasing concentrations of H_2_O_2_ resulted in modestly increased membrane deformability, as indicated by corresponding decrease in the shear modulus values (Fig. 1B). In order to ensure that the trend caused by H_2_O_2_ was not an artifact; RBCs were treated with higher concentrations (upto 30 mM) of the compound to yield a similar drop in the shear modulus (Supplemental: S2). This decline was, however, not significantly dose dependent beyond 3 mM concentration and yielded threshold values of *μ* = 4.96 µN/m (3mM H_2_O_2_), *μ* = 3.72 µN/m (10mM H_2_O_2_), *μ* = 3.30 µN/m (30mM H_2_O_2_) with a reference control value of *μ* = 7.52 µN/m (0mM H_2_O_2_). Further increase in H_2_O_2 _concentrations, resulted in cell lysis and formation of echinocytes.

### Changes in cytoskeletal architecture upon oxidative damage

**Figure 2:**
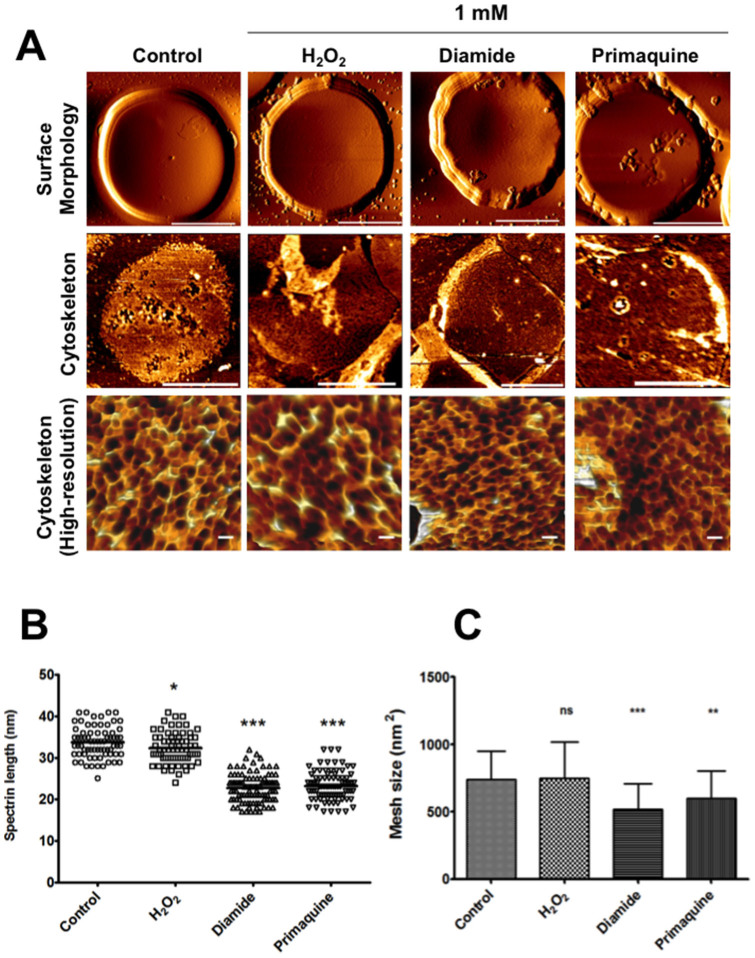
Oxidizing agents alters RBC membrane/cytoskeletal architecture. (A) Representative AFM images showing the surface morphology & cytoskeletal structures of oxidant-treated RBCs in comparison to non-treated controls. Outer surface images (2A: Top panel) were recorded in air contact mode, cytoplasmic surfaces were imaged in tapping mode (2A: Middle and Lower Panels). Height images were captured at a resolution of 256 × 256 pixels for whole cell images (Top Panel, whole cell scale bar: 4 μm) or 512 × 512 pixels for 1 µm × 1 µm areas for cytoskeletal structures, scale bar: 100 nm). (B) Average length of individual spectrin filaments observed from AFM images were quantified using ImageJ. Results indicated minor reduction in the length of spectrin fibers in H_2_O_2_- treated RBCs, while PQ and diamide resulted in significant reduction in spectrin length. (C) Average mesh sizes of spectrin networks observed from AFM images were quantified using ImageJ. Results showed no change in H_2_O_2_-treated RBCs whereas diamide and PQ significantly reduced the mesh sizes. *(p* value comparing the treatment vs control (ns: non-significant, * *p* <0.05, ** *p* <0.01, *** *p* <0.001, **** *p* <0.0001).

Oxidant exposure is known to cause Hb aggregation with red cell spectrin and lead to irreversible changes to the RBC cytoskeleton^5^ making them echinocytes. To qualitatively evaluate such cytoskeletal changes, we explored AFM imaging of the oxidant-treated cells of H_2_O_2_, diamide and PQ each at 1 mM concentration. We also conducted cytoskeletal imaging of cells exposed to CumOOH, but these cells did not adhere well to the coverslip according to the same protocol^9^ and were not analyzed. AFM imaging of the red blood cell's outer surface revealed differences that arose due to treatment with various oxidizing agents. Topographical images of RBCs (Fig. 2A, Top Panel) clearly demonstrate RBCs with the expected biconcave shape, except for PQ-treated ones. This observation is not surprising since PQ treatment changed many biconcave RBCs to cup-shaped stomatocytes (Fig. 1A), as described earlier. Analyses of the outer surface's fine structures showed no significant differences between H_2_O_2_ and non-treated controls. Both diamide and PQ treatments however resulted in cluster-like structures on the RBC surfaces- reflection of possible reorganization and aggregation of membrane components, induced by oxidant treatment.

To image the cytoskeletal structures from the inner cytoplasmic domain of the membrane, we analysed the cytoplasmic side of RBCs (Fig. 2A, middle & lower panels (high resolution)) as reported previously^9^. There were no detectable differences between non-treated cells and the cells treated with 1 mM H_2_O_2_. These images highlighted a series of criss-crossing ridges, forming hexagonal lattices representing normal spectrin array^9^,^21^,^22^. From the cytoskeletal images, average spectrin length (Fig. 2B) and mesh sizes (Fig. 2C) were estimated as reported previously^9^. Diamide and PQ treatments that resulted in reduced membrane deformability also appeared to decrease the average spectrin lengths and mesh size, thereby shrinking the cytoskeleton as evidenced from the AFM images. H_2_O_2 _treatment resulted in a minor reduction in the spectrin length but no change in mesh size.

### RBCs with stiffened membranes do not support plasmodial merozoite penetration

**Figure 3:**
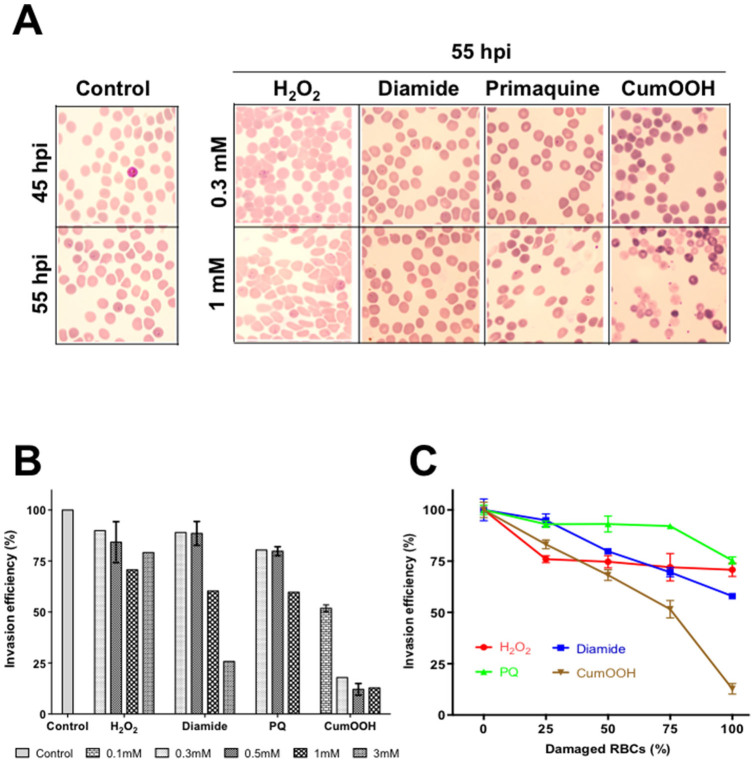
Oxidant-exposed RBCs are not preferred hosts for *P. falciparum* merozoites. (A) After treatment with selected concentrations of the four compounds, damaged RBCs were allowed to mix with purified schizont stage *P. falciparum* parasites and thin smears were investigated microscopically after staining with Giemsa. Plasmodial merozoites appeared to make initial contacts with red blood cells that were pre-treated with oxidizing agents, however failed to invade them except in the case of hydrogen peroxide treatment. (B) Invasion efficiency was expressed as percentage in comparison with control (untreated RBCs). (C) *P. falciparum* invades healthy RBCs more efficiently. RBCs were treated with 1 mM of various compounds, damaged RBCs (%) displayed mixture of 0, 25%, 50%, 75% and 100% damaged RBC to fresh RBCs. Invasion efficiency of each category was relative to the untreated RBCs. Data measured in triplicate and represented in mean ± SEM. Statistical significance was determined using a two-way analysis of variance, with *p* < 0.05 considered statistically significant.

Human malaria parasites tend to show distinct preferences towards host RBCs for infection. *Plasmodium (P.) vivax* and *P. ovale* invade young reticulocytes and *P. malariae* infect mature RBCs. *P. falciparum*, the most lethal human malaria variant, can invade RBCs of all ages^23^,^24^. However, there are reports indicating that *P. falciparum* may display mild preferential invasion, not specifically reticulocyte but relatively younger RBCs^25^,^26^. Several factors contribute to ageing of RBCs, exposure to oxidants being the major element. Hence, we evaluated the effect of oxidant-treatment on RBCs in relation to their ability to support invasion and growth of malaria parasites. To do this, RBCs pre-treated with oxidizing agents were washed and allowed to mix with purified infected RBCs (iRBCs) harboring late-stage *P. falciparum* parasites. Non-treated healthy RBCs were included as controls. Post-treatment, invasion ability of parasites to oxidant-treated red blood cells was evaluated through microscopic analyses of Giemsa-stained thin smears (Fig. 3A) and flow cytometric counting of ring-stage infections (Fig. 3B). Membrane stiffening due to oxidant exposure appeared to prevent merozoite penetration through the membrane/cytoskeleton specifically. The merozoites were clearly able to make initial contacts with the damaged red cells, however, they were found stuck on the red cell surface even 6 hrs after all the control parasites established ring-stage infections (Fig. 3A). All pre-treatments except for H_2_O_2_ significantly reduced the ability of plasmodial merozoites to invade in a dose-dependent manner (Fig. 3B). H_2_O_2_-treatment resulted in only a mild reduction (10–30%) in invasion efficiency.

To test the invasion efficiency of damaged RBCs in comparison to fresh RBCs, the intermediate concentration (1 mM) was selected for all four compounds for pre-treatment. Pre-treated RBCs were then mixed with fresh (non-treated) RBCs to obtain serial dilutions of 0, 25%, 50%, 75% and 100% damaged RBCs to fresh RBCs. Further, purified schizonts were added to individual samples and invasion was monitored. These results clearly showed the preference of merozoites towards healthy RBCs (Fig. 3C) compared to damaged ones. These results indicate likely roles of reactive oxidants in red cell ageing and its biological significance; such as plasmodial infectivity.

## Discussion

Here we investigated the morphological, mechanical and nano-structural changes occurring to oxidatively damaged RBCs, specifically to their membrane components, using a variety of techniques. These analyses collectively provide novel insights in to red cell morphology and membrane property changes in direct relation to biological consequences. We chose 4 unique oxidizing agents to induce damage to the red cells, H_2_O_2, _diamide, PQ and CumOOH, all of which have been used for similar studies before. H_2_O_2_ is thermodynamically unstable and decomposes to generate superoxide anions. In presence of cellular components such as hemoglobin as in the case of red blood cells, accelerated decomposition of H_2_O_2 _occurs initiating cellular damage. Diamide can induce oxidation of thiol groups and result in protein cross-linking, primarily RBC spectrins^27^. Although the mechanism remains largely unknown, PQ mediates oxidative stress by the generation of N-hydroxy and phenolic metabolites^28^. CumOOH generates alkoxyl and peroxyl radicals, which degenerate into uni-molecular radicals in presence of a catalyst like Hemoglobin^4^. Consequently this can result in spectrin-globin linkages or micro-vesciculation in the RBCs.

Effect of oxidizing agents on mechanical properties of erythrocytes has been explored previously, using methods such as ektacytometry and filterability on the population-wide whole cell deformability. Certain discrepancies are expected in such measurements since different experimental techniques measure different cellular responses – micropore filtration measures the ability of RBCs to pass through narrow openings, whereas ektacytometry measures the ability of RBCs to elongate when subjected to a well defined shear flow condition^4^,^5^,^7^. In other words, the whole cell response (influenced by membrane shear modulus, cell size, cell shape and cytosol viscosity) is taken into consideration, which can be different from the shear modulus of solely the membrane itself. Since ektacytometry utilizes a shear flow over the cells to measure the deformability, it is evident that changes in cell shape alone will result in changes in deformability measurements. The ektacytometry response generated by a discocyte is different from that of a stomatocyte even with the same membrane shear modulus, whereas the chosen technique of micropipette aspiration overcomes such non-specificity and provides the specific membrane deformability information. On the other hand the study carried out by thermal fluctuation measurements are dependent upon other factors such as ATP flux as acknowledged by the authors^4^ as well as cell shape. In addition this technique is not a direct measurement of the actual deformability of the RBC as compared to micropipette aspiration. The Micropipette aspiration on the other hand is a pressure driven system to interrogate the RBC membrane and consequently the shear resistance of the cytoskeleton.

Treatment with the oxidizing agents, except for H_2_O_2_, resulted in visible morphological changes in a dose-dependent manner. The red cell populations with significantly altered size and shape (primarily echinocytes and acanthocytes) do not represent classical bi-concave shaped RBCs. Thus, we specifically evaluated the bio-physical and nano-structural changes of only those RBCs that maintained the standard morphological characteristics of healthy RBCs. Previous literature suggests an overall reduction in red cell deformability upon oxidative damage^4^,^5^,^7^ caused by all compounds that we tested. Our results are in agreement with this trend for three of the compounds – Diamide, PQ and CumOOH. However, we noticed slightly increased RBC membrane deformability (specifically membrane shear modulus in this study) in response to H_2_O_2 _treatment, using micropipette aspiration technique. This apparent discrepancy may have arisen due to the distinctively different techniques used in the present study or as a consequence of the echninocyte populations that were possibly part of measurements using ektacytometry^5^ and micropore filtration measurements^6^ as reported earlier. Furthermore, there exist experimental evidences that peroxidation of the red cell lipid membrane could result in improved membrane fluidity and deformability, in the light of therapeutic use of ozone, a much more powerful oxidant than H_2_O_2_^29^. NOS-activating reagents have also been shown to improve RBC deformability and suggested as drugs for patients with diseases that have impaired RBC deformability^30^. Our results, therefore, do not necessarily contradict the previous findings^4^,^5^,^7^. They should rather be seen as complementary sets of information using an alternate technique that measures only membrane shear deformability. It also highlights the fact that there are different ways to measure RBC deformability focusing on different aspects (or combination of different aspects), and we would need to be careful in comparing the results obtained using different experimental methods. It is important to add independent data sets performed with different methods to supplement the available results in the literature in order to benefit from the strengths of the different techniques.

Representative AFM images exhibited visible differences on both extracellular and cytoplasmic surface of the membrane cytoskeleton at various conditions, especially on diamide and PQ-treated cells. These treatments induced significant aggregation of cytoskeletal components and might very well explain their reduced membrane deformability as observed from micropipette aspiration experiments. Quantitative evaluation of cytoskeletal meshwork damages was adopted to link the observed changes in membrane deformability and malaria infection efficiency with cytoskeletal nano-structural alterations. A protective effect against malaria infection is associated with inherited disorders in RBCs, such as cytoskeleton disorders, surface antigen gene mutations, enzymatic deficiencies or hemoglobin alterations^31^. Several earlier studies also reported the effect of erythrocyte membrane on the growth of malaria parasites^32^,^33^.

Malaria parasite invasion involves a number of mediators such as surface proteins that make contact with red blood cells, proteases that facilitates localized cytoskeletal re-organization and motor proteins to drive the parasites into the cell^34^. Our report establishes that damaged RBCs are more likely to be spared by *P. falciparum* merozoites during invasion, possibly due to a combination of biochemical, bio-physical and structural alterations arised due to oxidant exposure. However, we noticed that parasites were able to initiate the invasion process and recognize RBCs, hence it can be assumed that oxidant-treatment had no profound influence on the surface receptors and their presentation to allow parasite recognition. However, parasite entry into the red cells was affected, as altered cytoskeletal arrangement (shrinkage of structural network & increased stiffness and possible dehydration) could strongly influence the efficiency of various proteolytic enzymes involved in localized cytoskeletal re-modelling^35^ that drives parasite penetration into the red cells. These observations collectively demonstrate that the combination of morphological, bio-physical and cytoskeletal changes occurred during experimental oxidant-exposure make them less preferred hosts for malaria parasites in a similar fashion to natural ageing process of RBCs that happens *in vivo*, aged RBCs appear spared by P. falciparum.^25^,^26^ Taken together, this report provides new insights into the global alterations of human RBCs caused by different kinds of oxidative damages. The microstructural and membrane stiffness changes observed through micropipette aspiration and AFM imaging correlate significantly with the suitability of these cells to allow plasmodial parasite invasion, a cause of huge health concern to the developing world.

## Methods

### Ethics statement

All the experimental methods were carried out in accordance with the approved guidelines. The blood collection procedure was verified and approved by the Institutional Review Board (IRB) of National University of Singapore (NUS). All donors signed an informed consent for scientific research statement.

### Preparation of RBCs

Fresh blood samples from healthy donors were collected on a weekly basis in EDTA tubes (VACUETTE® EDTA Tubes, Greiner Bio-One) and stored for less than 2 h prior to processing. Blood was centrifuged at 600 × g for 10 minutes, after which buffy coat were removed and the remaining red blood cells (RBCs) were washed three times in RPMI 1640 (R8758 Sigma-Aldrich). Washed RBCs were stored in RPMI 1640 to a haematocrit of 50%. RBCs used in malaria culture were directly added to the culture flasks. RBCs used in oxidative stress assay were further washed twice in 1 × PBS (Vivantis, USA) containing 137 mM NaCl, 2.7 mM KCl and 10 mM Phosphate buffer, pH 7.4 and subsequently incubated with or without oxidizing agents as described below for further analyses.

### Treatments of RBCs with oxidative reagents

Oxidative stress was induced using the following agents: H_2_O_2 _(Merck Millipore), Diamide (Sigma-Aldrich), Primaquime bisphosphate (PQ) (Sigma-Aldrich) and Cumene hydroperoxide (CumOOH) (Sigma-Aldrich). Stock solutions were freshly prepared at 100 mM in PBS to make the required final concentrations as appropriate. Washed RBCs were exposed in PBS to oxidative agents at 2.5% haematocrit, in presence of 1% bovine serum albumin (BSA) (Miltenyi Biotec) to maintain the cell shape^4^,^5^. The experiment was carried out in 2 ml- tubes in a tube-revolver at 37°C for 1 h. Treated RBCs were washed three times in PBS to remove all traces of the compound and subsequently sent for analysis.

### DIC microscopy

Differential interference contrast (DIC) microscopy imaging was conducted using a suspension of RBCs maintained at RT (PBS + 1% BSA) after respective treatments. Images were recorded digitally using a 100 × immersion lens coupled with a 1.6 × magnifier by QColor5™. High Resolution Color CCD Digital FireWire™ Camera (Olympus I × 71 microscope) and processed by QCapture™ Pro 6.0. The maximum time span for each sample was 15 minutes.

### Micropipette aspiration

A borosilicate glass micropipette was used to extract the RBC membrane shear modulus (or membrane stiffness) by micropipette aspiration technique^8^. Pipettes were drawn from borosilicate glass tubing (Sutter Instrument Model P-2000) and cut open (Narishige MF-900) prior to mounting to the micromanipulator. The micropipette's inner diameter was approximately 1 ± 0.25 µm. A pressure drop rate of 1 Pa/s and a total pressure drop of 100 Pa were applied to aspirate and deform each cell. A total sample size of at least 20 cells per measurement was taken for individual experiments. The cell membrane was monitored by Olympus I × 71 microscope and processed by QCapture™ Pro 6.0 as described above. The maximum time span before the whole sample was replaced by a fresh sample was 1 h. From the high-resolution recordings, the leading edge of the aspired RBC membrane was tracked manually for calculating the elastic shear modulus using the Hochmuth model^8^.

### Sample preparation for atomic force microscopy

Thin smears were prepared imaging the outer surface of RBCs before and after treatment with oxidizing agents at preferred concentrations. Cytoplasmic-surface-exposed samples were prepared as described previously^9^. Briefly, coverslips (circle, φ 24 mm, Gerhard Menzel GmbH, Braunschweig, Germany) were first soaked in acetone, followed by 50% methanol/50% water to remove contaminants. Coverslips were then cleaned by plasma cleaner (Harrick Plasma, NY, USA) for 10 min. Cleaned coverslips were treated with 3-aminopropyl) triethoxysilane (APTES) (Sigma-Aldrich) for 3 min, rinsed in acetone for 5 min, followed by one time rinsing in 1 × PBS and finally in Mili Q water. APTES-treated coverslips were incubated with 150 µl of 1 mM Bis (sulfosuccimidyl) suberate (BS3) (Thermo Scientific) in PBS for 30 min and washed with PBS. This was followed by incubation with 150 µl of 0.1 mg/ml Erythroagglutinating phytohemagglutinin (PHA-E) (EY laboratories) solution for 2 h. Coverslips were subsequently rinsed with PBS three times and quenched with 0.1 M glycine for 15 min. After rinsing with PBS, the PHA-E coated cover slips were used readily for experiments or stored at 4°C for up to 2 weeks. Samples for imaging were incubated on the substrate for 3–4 h followed by rinsing with PBS. Cells were shear washed with at least 60 ml of 5P8- 10 buffer (5 mM Na_2_HPO_4_/NaH_2_PO_4_, 10 mM NaCl, pH 8.0) using a syringe at an angle of about 20 degrees. The cytoplasmic-surface-exposed-samples were vacuum dried overnight before AFM imaging.

### AFM imaging

JPK Nanowizard I AFM (JPK Instrument AG, Germany) was used to image both the smeared samples and cytoplasmic-surface exposed samples. Smear samples were imaged in air contact mode using MLCT probe (Bruker, Billerica, MA, USA). Super sharp silicon probes (SSS-NCHR probes, Nanosensor, Neuchatel, Switzerland) with tip radius of about 2 nm were used to image RBC cytoskeleton in tapping mode^9^. Height images were captured at a resolution of 256 × 256 pixels for 10 µm × 10 µm or 512 × 512 pixels for 1 µm × 1 µm areas. Individual spectrin filaments and mesh were analyzed using ImageJ for estimating the average lengths and sizes^9^.

### Parasite culture and malaria invasion experiments

*Plasmodium falciparum* (3D7) parasites were maintained under standard condition as described previously^36^. Parasites were synchronized by sorbitol selection of rings. To test the effect of different oxidative agents on parasite invasion, fresh RBCs were exposed to each compound at required concentrations. Treated RBCs were then plated at 2.5% hematocrit in culture medium (RPMI 1640/GIBCO BRL supplemented with HEPES, hypoxanthine, sodium bicarbonate and Albumax II). Purified schizonts (> 40 h post invasion) were isolated by magnetic selection^37^ (MACS Miltenyi Biotec, Germany) and mixed with damaged RBCs to obtain approximately 1% parasitemia and allowed to invade. Parasitemia was assessed after 16 h by microscopy and flow cytometry.

To test the invasion efficiency of parasites on damaged RBCs in comparison to fresh RBCs, the intermediate concentration (1 mM) was selected for all four compounds. After 1 h incubation with different oxidative agents, RBCs were washed three times in RPMI 1640. These pre-treated RBCs were then mixed with fresh (non-treated) RBCs to obtain serial dilutions of 0%, 25%, 50%, 75% and 100% damaged RBC to fresh RBCs (final hematocrit 2.5% in culture medium). Each sample was then allowed to mix with purified schizonts and parasitemia were determined by flow cytometry and Giemsa-staining 16 h later.

### Flow cytometry

Flow cytometric was performed on a BDTM LSRII flow cytometer (Becton, Dickinson, CA. USA), data acquisition and analysis were done using FlowJo v8.8.7 (Tree Star, Inc., Ashland, OR). At least, fifty thousand events were analysed for each treatment. For determining parasitemia^38^, 100 µl aliquots of parasites from respective wells were collected and fixed with 0.1% glutaraldehyde (Sigma-Aldrich) at 4°C overnight. Fixed cells were collected by centrifugation (600 g, 5 min), washed in PBS, permeabilized using 0.25% Triton X-100/PBS (Sigma-Aldrich) for 5 min at RT and washed. Further, samples were incubated with 25 µg/ml Hoechst 3342 for 30 min sheltered from light, washed twice in PBS and subsequently counted by flow cytometery. For Hoechst detection, samples were excited by 355 nm UV laser. Initial gating was carried out with unstained, uninfected erythrocytes to account for auto-fluorescence. Non-treated RBCs included in the same experiments served as controls for gating. Parasitemia counts were verified by comparing to corresponding Giemsa- stained smears.

### Statistical analysis

GraphPad Prism (GraphPad Software Inc, USA) was used for analysing the statistical significance of damage effect caused by different oxidative agents. Micropipette data are expressed as mean ± range unless mentioned otherwise. A preliminary analysis of variance (one-way) was performed to test differences in mean values within; before and after treatment. Data on malaria invasion are presented as Mean ± SEM, with, p <0.05 considered statistically significant.

## Author Contributions

A.S.: Involved in study design, conducted experiments and assisted in manuscript preparation; T.C.: Involved in study design, conducted experiments and assisted in manuscript preparation; M.D.: Involved in study design, provided tools for research, verified results and assisted in manuscript preparation; R.C.: Designed & coordinated research, verified results and wrote the manuscript.

## Supplementary Material

Supplementary Information Supplemental Information
