# Supplementary material for: Single-cell evaluation of red blood cell bio-mechanical and nano-structural alterations upon chemically induced oxidative stress
Source: Sci Rep. 2015 May 7;5:9768. doi: 10.1038/srep09768 (PMC4423428; doi:10.1038/srep09768)
Supplement: Supplementary Information — Supplemental Information [file srep09768-s1.pdf]

Single-cell evaluation of red blood cell bio-mechanical and nano-structural alterations upon chemically induced oxidative stress

Ameya Sinha[¥#¶], Trang TT Chu[¥¶], Ming Dao[#†*] and Rajesh Chandramohanadas[¥*]

Table S1. Statistics related to the micropipette analyses.

Fig. S2. RBCs treated with higher H_2O_2 concentrations resulted in increased deformability. Data is represented as median ± range.

Table S3. Statistical assessment of the AFM images to estimate spectrin length.

Supplemental: S1

Table S1.
Statistical assessment of the Micropipette data: Deformability

H_2O_2	Control	0.3mM	1mM	3mM
Mean (μN/m)	8.888	7.515	6.613	6.054
Standard Deviation	1.702	1.73	1.336	0.8793
Standard Error of Mean	0.3805	0.3868	0.2987	0.1966
**Diamide**	**Control**	**0.3mM**	**1mM**	**3mM**
Mean (μN/m)	7.107	9.615	11.83	14.92
Standard Deviation	1.76	1.319	2.847	5.045
Standard Error of Mean	0.4546	0.3406	0.7352	1.303
**Primaquine**	**Control**	**0.3mM**	**1mM**	**3mM**
Mean (μN/m)	4.19	5.266	6.503	10.94
Standard Deviation	0.5098	1.662	1.688	3.579
Standard Error of Mean	0.114	0.3717	0.3774	0.8003
**CumOOH**	**Control**	**0.1mM**	**0.3mM**	**1mM**
Mean (μN/m)	5.779	6.949	13.2	-
Standard Deviation	1.375	1.712	4.623	-
Standard Error of Mean	0.355	0.442	1.194	-

H_2O_2	Control	3mM	10mM	30mM
Mean (μN/m)	7.522	4.961	3.722	3.297
Standard Deviation	2.199	2.095	1.396	0.8685
Standard Error of Mean	0.5678	0.5409	0.3605	0.2242

Supplemental: S2

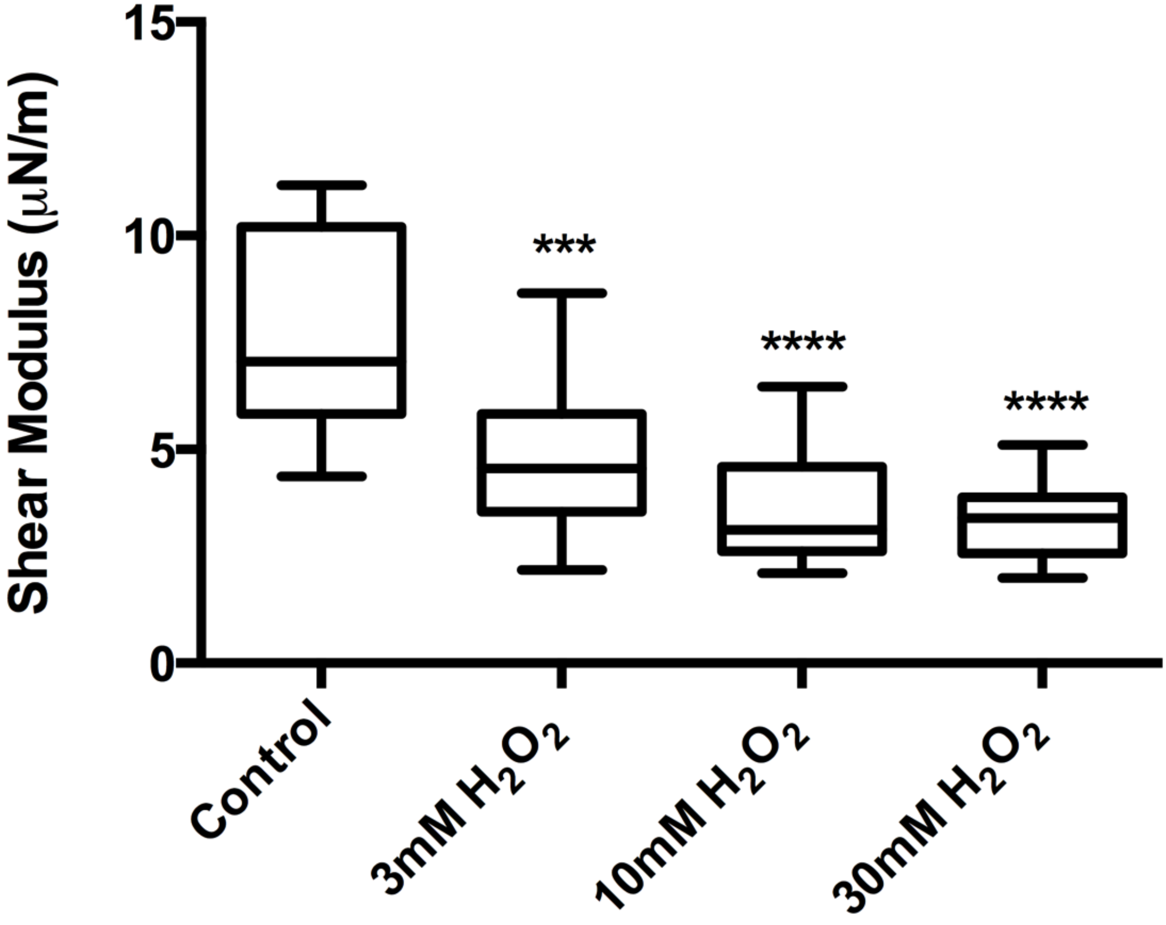

Supplemental: S3

Table S3.
Statistical assessment of the AFM data: spectrin length

	Control	H_2O_2	Diamide	Primaquine
**Mean (nm)**	34.5	32	22.7	23.2
**Standard Deviation**	4.47	3.86	3.41	3.48
**Standar Error of Mean**	0.4682	0.3856	0.3378	0.3727
**Number of values**	91	100	102	87
